# LPS Regulates SOCS2 Transcription in a Type I Interferon Dependent Autocrine-Paracrine Loop

**DOI:** 10.1371/journal.pone.0030166

**Published:** 2012-01-23

**Authors:** Jin Hu, DaoHua Lou, Berit Carow, Malin E. Winerdal, Martin Rottenberg, Ann-Charlotte Wikström, Gunnar Norstedt, Ola Winqvist

**Affiliations:** 1 Translational Immunology Unit, Department of Medicine, Karolinska Institutet, Stockholm, Sweden; 2 Department of Molecular Medicine and Surgery, Karolinska Institutet, Stockholm, Sweden; 3 Department of Biosciences and Nutrition, Karolinska Institutet, Stockholm, Sweden; 4 Department of Microbiology, Tumor and Cell Biology, Karolinska Institutet, Stockholm, Sweden; 5 Department of Trauma Orthopedic and Hand Surgery, The First Affiliated Hospital of Guangxi Medical University, Nanning, People's Republic of China; Charité, Campus Benjamin Franklin, Germany

## Abstract

Recent studies suggest that SOCS2 is involved in the regulation of TLR signaling. In this study, we found that the expression of SOCS2 is regulated in human monocyte-derived DC by ligands stimulating TLR2, 3, 4, 5, 8 and 9 signaling. SOCS2 induction by LPS was dependent on the type I IFN regulated transcription factors IRF1 and IRF3 as shown by using silencing RNAs for IRFs. Blocking endogenous type I IFN signaling, by neutralizing antibodies to the receptor IFNAR2, abolished SOCS2 mRNA expression after TLR4 stimulation. Transcription factors STAT3, 5 and 6 displayed putative binding sites in the promoter regions of the human SOCS2 gene. Subsequent silencing experiments further supported that STAT3 and STAT5 are involved in LPS induced SOCS2 regulation. In mice we show that SOCS2 mRNA induction is 45% lower in bone marrow derived macrophages derived from MyD88^−/−^ mice, and do not increase in BMMs from IRF3^−/−^ mice after BCG infection. In conclusion, our results suggest that TLR4 signaling indirectly increases SOCS2 in late phase mainly via the production of endogenous type I IFN, and that subsequent IFN receptor signaling activates SOCS2 via STAT3 and STAT5.

## Introduction

Antigen-presenting cells (APCs) are able to recognize microbes based on pattern-recognition receptors such as Toll-like receptors (TLRs). The TLR family is widely expressed among inflammatory cells and includes 11 members in humans and 13 in mouse [1], [2]. Each TLR recognizes different microbial molecules resulting in the recruitment of cytoplasmic adaptors to their Toll/IL-1 receptor (TIR) domain and subsequent activation of cellular programs [2], [3]. There are two major independent but complementary pathways in TLR signaling: (I) the MyD88-dependent pathway, which recruits the adaptor MyD88 upon TLR2, 4, 5, 7, 8 and 9 activation or MyD88-adaptor like (MAL) upon TLR2 and 4 activation. The MyD88 dependent activation leads to NFκB, AP-1, IFN regulatory factor 5 (IRF5) and IRF7 nuclear translocation that controls the expression of inflammatory cytokine genes such as TNFα, IL-1β and IL-12. (II) The MyD88-independent pathway which induces the recruitment of the TIR domain-containing adaptor (TRIF) upon TLR3 and 4 activation and the TRIF related molecule (TRAM) adaptor upon TLR4 activation, leading to IRF3 nuclear translocation inducing the expression of mainly type I IFN and IFN-inducible genes [4], [5]. Recently, more members of the IRF family, IRF1 [6], IRF7 [7] and IRF8 [8], have been demonstrated as important transcriptional factors for the induction of type I IFN.

Evolution has developed several lines of negative regulation mechanisms to keep TLR and ensuing inflammatory responses at adequate levels. The involved negative regulators are divided into 2 groups: signal-specific regulators that inhibit signal transduction by TLRs such as SOCS proteins and gene-specific regulators that function to modulate gene expression [9]. The members of SOCS family consisting of SOCS1-7 and cytokine-inducible Src homology 2 protein (CIS) have been found to negatively regulate JAK-STAT signaling. SOCS1 and 3 have been also shown to modulate TLR4 signaling [10]. SOCS1 interacts with phosphorylated MAL resulting in its polyubiquitylation and subsequent degradation by the proteasome [11]. In addition SOCS1 and SOCS3 also inhibit NF-κB activation and thereby regulate TLR4 signaling [12].

SOCS2 is a well established negative regulator of growth hormone (GH) signaling via the JAK/STAT pathway [13] and docks to the intracellular domains of related receptors or facilitates proteasome-dependent degradation of transcription factors [14]. Recently, the action of the anti-inflammatory drug, acetylsalicylic acid, was shown to be SOCS2-dependent, indicating an important role of SOCS2 in the regulation of infectious and inflammatory responses [15]. Furthermore, the HIV-1 transactivator protein Tat, one of the retroviral proteins identified as a key immunomodulator in the pathogenesis of AIDS, interfered with the IFN-γ receptor signaling pathway at the level of STAT1 activation, possibly via Tat-dependent induction of SOCS2 activity induced by HIV infection, again pointing towards SOCS2 as an important modulator of immune responses [16].

SOCS2 has been shown to be induced by the TLR2 ligand LXA4 in mouse splenic DCs [15] and the TLR4 ligand LPS in human DCs [17]. However, the regulation of SOCS2 expression by inflammatory stimuli in the cells of immune system has not been extensively studied. In contrast, more in depth studies have been performed on SOCS2 transcription in GH signaling. GH signaling leads to SOCS2 transcription via induction of the transcription factor STAT5b. A novel response element for STAT5b was identified within the first intron of the human SOCS2 gene, composed of an E-box followed by tandem STAT5b binding sites, both of which are required for full GH responsiveness [18]. We previously reported that SOCS2 is substantially induced by LPS stimulation in human monocyte derived dendritic cell (moDCs) [17]. In this study, we further investigate the transcriptional regulation of SOCS2 expression in TLR4 signaling.

## Results

### TLR ligands induce SOCS2 gene expression in human moDCs

**Figure 1:**
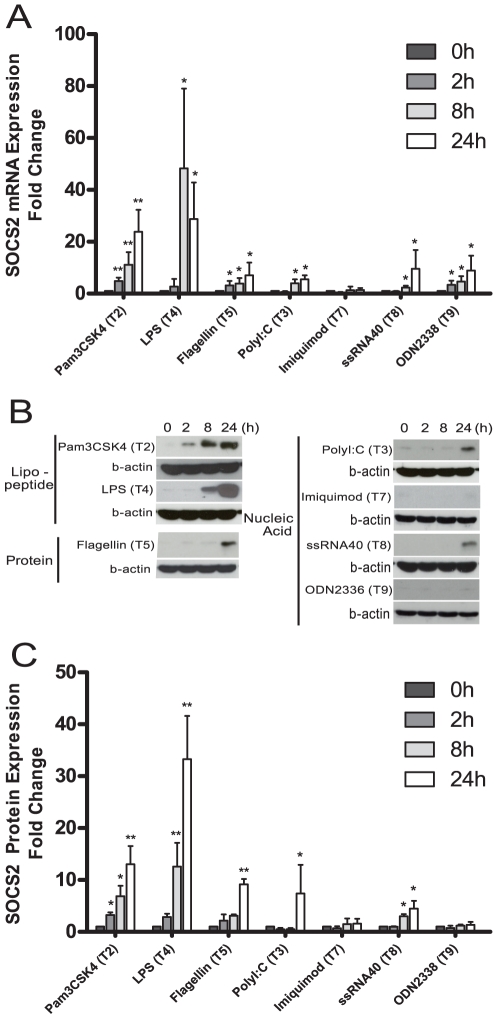
Various TLR ligands induce SOCS2 gene expression in human moDCs. (A). Human SOCS2 mRNA expression induced by different TLR signaling. Enriched monocytes were cultured in the presence of GM-CSF and IL-4 for 6 days. Quantitative real-time PCR was used to measure SOCS2 mRNA expression in iDC after exposure to Pam3CSK4 (TLR1/2), LPS (TLR4), flagellin (TLR5), polyI:C (TLR3), imiquimod (TLR7), ssRNA40 (TLR8) and ODN 2336 (TLR9) for different time periods. Data shown are the mean of triplicate determinations from at least three donors. Values for the time point 0 were set to 1. (B, C). SOCS2 protein expression levels after various TLR signaling stimuli. Western Blotting analysis for protein of SOCS2 expression in iDCs exposed to Pam3CSK4 (TLR1/2), LPS (TLR4), flagellin (TLR5), polyI:C (TLR3), imiquimod (TLR7), ssRNA40 (TLR8) and ODN 2336 (TLR9) for different time points. (B) Representative Western Blot for each typical TLR signaling effect to SOCS2 expression. The data shown in (C) are the mean of SOCS2 protein bands quantified from Western blots and normalized to β-actin from four donors. Values for time point 0 were set to 1. Error bars comparing different time point groups to the 0 time point group illustrate s.d. * p<0.05; ** p<0.01.

Toll-like receptors recognize microbial patterns, and can be arranged into three major families by their ability to bind lipids (TLR2 and TLR4), proteins (TLR5) and nucleic acids (TLR 3, 7, 8 and 9) [4]. We first investigated the ability of different TLR agonists to modulate SOCS2 expression in APCs. Human immature DCs (iDCs) were obtained by differentiating monocytes with GM-CSF and IL-4. On day6 the cells were stimulated with various TLR ligands including Pam3CSK4 (TLR2), LPS (TLR4), flagellin (TLR5), polyI:C (TLR3), imiquimod (TLR7), ssRNA40 (TLR8) and ODN2336 (TLR9). Real-time PCR analysis demonstrated significantly increased expression of SOCS2 mRNA levels 8 h to 24 h after stimulation by TLR2, TLR3, TLR4, TLR5, TLR8 and TLR9 binding ligands (Figure 1A). Interestingly, stimulation by Pam3CSK4 and LPS resulted in the highest SOCS2 mRNA accumulation (23 and 48 fold increase). The SOCS2 induction was moderate when iDCs was stimulated by either flagellin (7 fold), polyI:C (6 fold), ssRNA40 (10 fold) or ODN 2336 (9 fold). SOCS2 mRNA level in DCs stimulated with TLR7 agonist was not increased (Figure 1A). This was partially expected with regard to TLR 7 signaling, since TLR7 has not so far been reported to be expressed in human DCs [19] whereas the expression has been demonstrated in mouse myeloid DCs [20].

SOCS2 protein levels were also increased in DCs incubated with Pam3CSK4, PolyI:C, LPS, flagellin and ssRNA40 treatment (Figure 1B) corresponding to the changes seen in the increased SOCS2 mRNA expression levels (Figure 1A). The increased SOCS2 protein expression became obvious at 8 h and increased dramatically up to 24 h. However, ODN 2336, the ligand for TLR9, did not induce increased SOCS2 protein levels (Figure 1B right panel) in contrast to results at the transcriptional level where a 10 fold increased expression of SOCS2 message was demonstrated (Figure 1A). Quantification of SOCS2 protein expression on stimulated moDCs from different healthy blood donors demonstrated a good correlation with the mRNA expression data with the exception of TLR9 signaling. We conclude that TLR signaling stimulates SOCS2 expression, with the dominant inducer being LPS.

### Type I IFN regulated transcription factors are involved in SOCS2 induction

**Figure 2:**
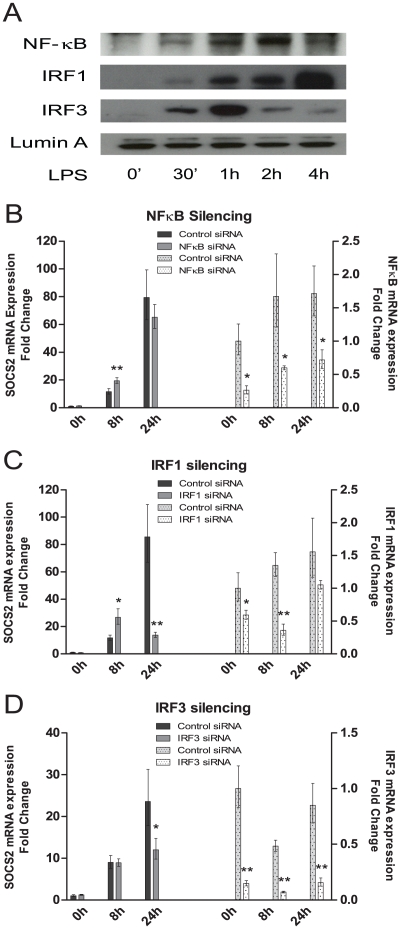
Type I IFN regulated IRFs, but not NF-κB were required for SOCS2 mRNA induction by TLR4 signaling in human moDCs. (A) NF-κB, IRF1 and IRF3 nuclear translocation in TLR4 signaling in human moDCs. moDCs were stimulated with LPS at the indicated time points. Nuclear protein was extracted for Western Blotting measurement. The levels of p65NF-κB, IRF1, IRF3 and Lamin A (as loading control) were detected. (B–D) The effect of silencing NF-κB, IRF1 and IRF3 on the induction of SOCS2 mRNA in LPS stimulated human moDCs. moDCs were transfected with (B) NF-κB, (C) IRF1, (D) IRF3 siRNAs or control siRNA and incubated for 24 hours. The transfected cells were exposed to LPS for 0 h, 8 h or 24 h, the cells were then harvested for the mRNA measurement by qRT-PCR to demonstrate the effect of SOCS2 mRNA induction (left part) and efficiency of target gene knockdown (right part). Data shown are representative of three independent experiments and are expressed as the –fold induction of the gene of interest at different time points compared to time point 0 for control siRNA where the values were set to 1. Statstical significance comparing results from the target siRNA to control siRNA group is indicated (* p<0.05; ** p<0.01).

Since LPS was found to be the main inducer of SOCS2 expression (Figure 1) we next studied SOCS2 transcriptional regulation by TLR4 signaling comprehending both MyD88-dependent and -independent pathways [4]. In conventional DCs (cDCs), LPS activates NF-κB as a part of the MyD88-dependent pathway and IRF3, which regulates type I IFN transcription in a MyD88-independent manner. Recently, also IRF1 has been shown to have a role in type I IFN induction during activation of the MyD88-dependent pathway [6]. iDCs were stimulated with LPS and transcription factors involved in TLR4 signaling were measured in nuclear fractions. P65NF-κB, IRF1 and IRF3 translocated from the cytoplasm to the nucleus after 30 minutes to 1 h. Interestingly, NF-κB and IRF3 nuclear translocation peaked around 1 h whereas IRF1 translocation peaked after 4 h (Figure 2A).

In order to identify the transcriptional factors involved in SOCS2 expression, we silenced p65NF-κB, IRF1 and IRF3 in human moDCs with siRNA, and then stimulated the transfected cells with LPS. Compared to control siRNA, all tested siRNAs demonstrated an efficient knock-down of target gene expressions. The range of knock-down efficiency was from 33 to 85% (figure 2B–D right parts). Surprisingly, p65NF-κB knock-down had no effect on SOCS2 induction (Figure 2B, left part). However, IRF1 (Figure 2C, left part) and IRF3 (Figure 2D, left part) silencing decreased SOCS2 induction significantly at the 24 h time point. We conclude that IRF1 and IRF3 pathways mediate SOCS2 expression.

### Type I IFN is required for SOCS2 induction

**Figure 3:**
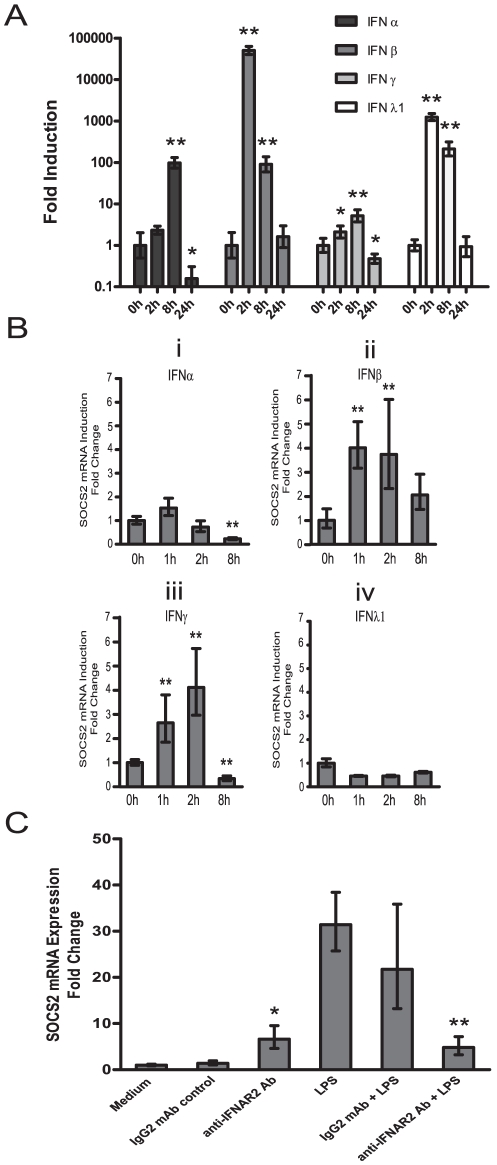
The effect of type I IFNs to SOCS2 mRNA induction in human moDCs. moDCs were exposed to LPS (A) for indicated time periods for mRNA measurement of IFNα, IFNβ, IFNγ and IFNλ1 or incubated with IFNα (Bi), IFNβ (Bii), IFNγ (Biii) and IFNλ1 (Biv) for indicated time periods for SOCS2 mRNA measurement by qRT-PCR. (C) moDCs were pretreated for 30 min with or without 30 ug/ml neutralizing anti-IFNR2 antibodies or IgG_2a_ isotype control mAb and then incubated for 4 h with or without LPS for SOCS2 mRNA measurement by qRT-PCR. Data shown are representative of three independent experiments and expressed as the –fold induction of the gene of interest at different time points compared to time point 0 where the values were set to 1(A–B) or different treatment conditions compared with medium that values were set to 1. Statistical significance of results with anti-IFNAR2 mAb compared with control mAb is indicated (C) (* p<0.05; ** p<0.01).

Since the IFN response factors IRF1 and IRF3 regulated SOCS2 expression we studied the role of IFNs as inducers of SOCS2. The IFNs are classified into type I IFNs including α, β etc more than 20 other subtypes, the single type II IFNγ and three type III IFNλs [21], [22]. We first investigated which type of IFNs that might be involved in SOCS2 induction after TLR4 signaling in human moDCs. After LPS treatment, we measured IFNα, IFNβ, IFNγ and IFNλ1 mRNA expression at different time points. After 2 h, IFNβ and IFNλ1 mRNA were induced dramatically, the levels were maintained until 81h and returned to base line after 24 h (Figure 3A). IFNα showed a similar profile except a later induction at 8 h instead of 2 h, whereas IFNγ was only moderately induced after LPS treatment (Figure 3A). These results are consistent with previous findings [23].

To further clarify which IFN are able to induce SOCS2 expression, we treated moDCs with IFNα, IFNβ, IFNγ or IFNλ1 respectively, and measured SOCS2 mRNA expression. IFNα induced a small increase in SOCS2 expression (Figure 3Bi). In contrast, both IFNβ and IFNγ showed a rapid and substantial induction of SOCS2 expression after 1 h stimulation (Figure 3Bii–iii). IFNλ1 did not induce any SOCS2 increase (Figure 3Biv). Taken together LPS induced IFNβ and IFNβ has the direct ability to stimulate SOCS2 expression in human moDCs. Though SOCS2 was induced around 5 folds after IFNβ stimulation (Figure 3Bii) but almost 50 folds in LPS treatment (Figure 1A), this obvious difference of SOCS2 induction was most likely a consequence that sustained LPS stimulation induces an IFN-dependent amplification loop causing a production of large amount of IFNβ continuously. To further prove that endogenous type I IFN production has a role for SOCS2 induction, we added a neutralizing anti-human interferon-α/β receptor (IFNAR) 2 antibody before LPS activation of moDCs. The effect of IFNAR2 blocking was investigated after 4 h of stimulation, since our previous data show that LPS induce a significant increase in SOCS2 mRNA expression at that time point [17]. Addition of anti-human IFNAR2 antibody blocked the SOCS2 mRNA induction in response to LPS treatment, whereas incubation with the isotype control antibody did not (Figure 3C). These suggest that endogenous type I IFN mediates SOCS2 expression in response to LPS.

### Transcription factors STAT3, 5 and 6 are translocated to the nucleus in response to LPS and IFNβ

The TLR4 ligand LPS is a well known inducer of acute inflammation. In APCs, several hundred genes are induced within a few hours of LPS stimulation [24]. Dedicated transcription factors coordinately regulate these gene sets or transcriptional modules. The genes induced by TLR4 signaling can be grouped into three classes based on time lapse after LPS stimulation. The primary response genes are induced within 0.5–2 h, the secondary response genes are induced after 2–8 h, and specific gene expression is induced after a longer period of time [25]. Since SOCS2 mRNA levels increased significantly after 4 h LPS stimulation [17] and within 1 h after IFNβ stimulation (Figure 3Bii), we hypothesized that SOCS2 was induced by type I IFN as a secondary response gene after LPS stimulation.

STATs are known as essential components of the type I IFN receptor signaling cascade [21], [26]. Therefore we decided to study the human SOCS2 promoter region and search for potential binding sites for STAT family members. Three promoter regions (Figure S1A) in the SOCS2 gene were investigated. We found several putative binding sites for STATs in the promoter regions 1, 2 and 3 (As shown in Figure S1B); STAT5 in the promoter region 1, STAT3 in the promoter region 2 and STAT3, 5, 6 in the promoter region 3. Thus, STAT3, 5 and 6 were identified as putative SOCS2 regulating transcriptional factors for type I IFN signaling in human DCs. Therefore we addressed the role of these transcription factors in SOCS2 induction in our experimental system.

**Figure 4:**
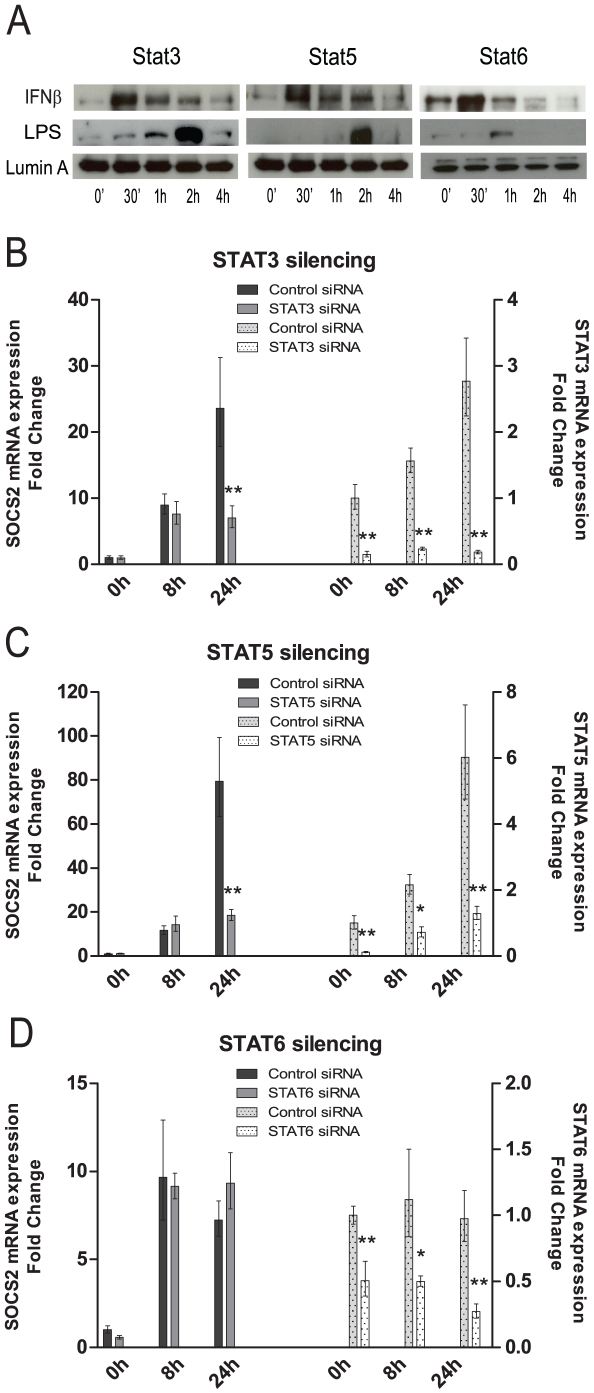
STAT3 and STAT5, but not STAT6 are required for SOCS2 mRNA induction in human moDCs. (A) STAT3 and STAT5, but not STAT6 were translocated into the nucleus after IFNβ or LPS stimulation. moDCs were incubated with IFNβ or LPS at the indicated time points. The cells were then harvested and nuclear proteins were extracted for Western Blotting measurement. The levels of STAT3, STAT5, STAT6 and LaminA (as loading control) were detected. (B, C and D) STAT3 and STAT5, but not STAT6 gene silencing eliminates SOCS2 mRNA induction in TLR4 signaling. moDCs were transfected with (B) STAT3, (C) STAT5 or (D) STAT6 siRNAs or control siRNA and incubated for 24 hours. The transfected cells were exposed to LPS for 0 h, 8 h and 24 h, the cells were then harvested for the mRNA measurement by qRT-PCR to demonstrate the effect of SOCS2 mRNA induction (left) and efficiency of target gene silencing (right). Data shown are representative of three independent experiments and expressed as the –fold induction of the gene of interest at different time points compared to time point 0 for control siRNA where values were set to 1. Significance comparing results from the target siRNA to the control siRNA group is indicated (* p<0.05; ** p<0.01).

If STATs are transcriptional factors regulating SOCS2 induction after type I IFN signaling, i.e. the subsequent signaling cascade of TLR4 signaling, the STATs should be activated after type I IFN or LPS stimulation in a time correlated way. To prove this, nuclear proteins were extracted from LPS or IFNβ treated iDCs at different time points and the nuclear translocation for the predicted STAT3, 5 and 6 transcriptional factors was demonstrated by Western Blot. The STAT3, 5 and 6 transcription factors were all translocated into the nucleus 30 minutes after IFNβ stimulation (Figure 4A). However, the LPS induced translocation occurred at 2 h for STAT3 and STAT5, and only weakly for STAT6 at 1 h (Figure 4A). Thus, LPS induction of SOCS2 is most likely dependent on IFNβ and its subsequent activation of STAT3 and STAT5.

### Transcription factors STAT3 and STAT5 are required for SOCS2 induction

To further determine the role of predicted SOCS2 promoter region binding transcription factors STAT3, STAT5 and STAT6 for the TLR4 signaling induction of SOCS2, we performed gene silencing experiments using STAT3, STAT5 and STAT6 siRNAs to check their effect on SOCS2 mRNA expression. When STAT3 expression was silenced, a significant reduction (>70% decrease) of LPS induced SOCS2 mRNA expression was observed (Figure 4B). A similar effect was found in STAT5 silenced moDCs (>76% decrease) (Figure 4C). Consistent with our results of a weak STAT6 nuclear translocation after LPS stimulation (Figure 4A) successful silencing of STAT6 did not affect SOCS2 mRNA expression (Figure 4D). Thus, Stat3 and Stat5 are most likely the main transcription factors regulating SOCS2 induction after TLR4 signaling.

### The MyD88–independent signaling is the major pathway involved in SOCS2 induction in mouse bone marrow derived macrophages

To verify our findings in a more physiologically relevant situation we studied BCG responses of bone marrow derived macrophages (BMM) from MyD88^−/−^ and IRF3^−/−^ mice. BCG stimulates TLR signaling, mainly by engaging TLR2 and TLR4 receptors followed by subsequent activation of MyD88-dependent and -independent pathways, causing NF-κB and IRF3 nuclear translocation [34], [35].

**Figure 5:**
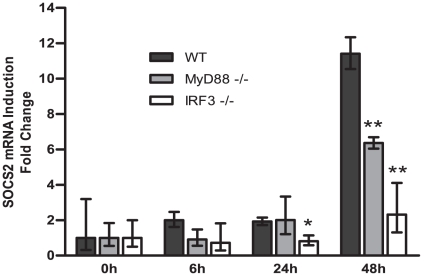
IRF3, but not MyD88, mainly involved in SOCS2 mRNA expression after BCG infection in mice bone marrow macrophages (BMM). BMM from wild-type mice, MyD88 ^−/−^ mice or IRF3 ^−/−^ mice were activated with BCG for indicated time periods, the cells were then harvested for SOCS2 mRNA measurement by qRT-PCR. Data shown are the mean of triplicate determinations from three independent experiments. Values for time point 0 were set to 1. Significance comparing results from the deficient mice to wild-type mice is indicated (* p<, 0.05; ** p<, 0.01).

In BMM from wild type mice, SOCS2 mRNA was induced 48 h after BCG infection (Figure 5). Unlike when tested in human moDCs (Figure 2B), SOCS2 mRNA expression was abrogated about 45% in BMM from MyD88^−/−^ mice. However, SOCS2 induction was almost completely eliminated in BMM derived from IRF3^−/−^ mice (Figure 5). These results demonstrate that SOCS2 induction after BCG engagement is dependent on the IRF3/IFN pathway and that both MyD88-dependent and –independent pathways are involved.

## Discussion

In this report, we demonstrate that expression of SOCS2 in human moDCs is mediated by “endogenous” type I IFN signaling after LPS treatment. SOCS2 is stimulated in a relatively slow fashion, and inhibition of the IFNAR2 receptor signaling strongly reduced the SOCS2 expression. Thus, although the promoter region of SOCS2 contains a consensus sequence for IRF3 binding (not shown), it is unlikely that direct IRF3 binding to the promoter region of SOCS2 is an important regulatory mechanism.

Type I IFN has been shown to play an important role in the cascade of gene expression after TLR signaling [22], [24]. In particular, TLR4 has been demonstrated to induce synthesis and secretion of IFNβ by APCs that secondarily stimulate the expression of IFN-regulated genes [23], [27] and in addition negative regulators to adjust signaling [28]. Our data suggest that an autocrine/paracrine type I IFN loop is required for LPS to stimulate SOCS2 expression (Figure S2). A recent publication suggested that SOCS2 is a direct downstream target of TLR ligation [29]. However, the effect of IFN to SOCS2 induction was not investigated. We clearly demonstrate that the inhibition of IRF1, IRF3 and INFAR2 signaling severely impairs SOCS2 induction (Figure 2 and 3C). Though the siRNA experiments in Figure 2 showed the effect of IRF1 and IRF3 for SOCS2 induction at the 24 h time point and the experiment in figure 1 using LPS activation of DCs responded already after 8 h, the different experimental settings between the figures likely affected the time courses. We have previously observed similar effect caused likely by siRNAs transfection process [29]. In contrast to IRFs, NF-κB silencing only marginally affected SOCS2 expression in our study. This data demonstrates the importance of type I IFN for the LPS induced SOCS2 in an indirect loop way.

The importance of IFN signaling for SOCS2 regulation was further supported in IRF3 and MyD88 deficient mice experiments. While MyD88 signaling only partially affects SOCS2 induction, SOCS2 induction was almost completely abolished in IRF3 deficient macrophages (Figure 5). Compared to the former human siRNAs knock-down experiments, SOCS2 expression was induced in mice macrophages after 48 h BCG infection. It is most likely caused by the different experimental systems including species (human to mice), cells used (DCs to macrophages) and stimulators (LPS to BCG). However, these also indicate that SOCS2 expression is mainly induced in the late phase of infection in an IFN-mediated manner.

IFNAR activation in the presence of LPS stimuli leads to translocation of STATs, particularly STAT3 and STAT5 (Figure 4) followed by increased SOCS2 expression. Both IFNα- and IFNβ-dependent signals can activate STATs [21]. Stat5 is phosphorylated on serines 725/730 and this is required for type I interferon-dependent gene transcription via gamma interferon activation site (GAS) elements [30]. STAT5 has previously been demonstrated by our group to regulate mouse and human SOCS2 genes via binding to their promoter regions [18]. In agreement, binding sites of STAT5 in the human SOCS2 gene can be predicted (Figure S1) and may explain how SOCS2 is activated by IFNβ in human cells. Consistent with previous studies [29], we show that STAT3 is activated by IFNβ and is required for TLR-dependent SOCS2 expression. This finding may contribute to the understanding of the role of STAT3 activation in rendering DCs ineffective [31].

The present study as well as a previous report [29] show that signaling via other TLRs besides TLR4 increase SOCS2 expression. This may suggest that increased SOCS2 is of importance for the control of host defense, i.e. by regulating TLR signaling. TLR3, 8 and 9 agonists all enhance type I IFN secretion by human DCs [6], [32], [33], providing a plausible explanation for SOCS2 induction by those TLRs signals (Figure 1). Interestingly, we found that TLR2 and TLR5 activation, which do not activate IFN secretion, also induced SOCS2 expression in our study. It implied that besides IFN signaling additional signals may be involved, and may also explain why MyD88^−/−^ BMM partly affect SOCS2 expression after BCG infection (Figure 5).

The functional consequence of TLR activation of SOCS2 is incompletely understood. Though the SOCS family has been demonstrated to play a role as negative cytokine signaling regulators, the data from SOCS deficient mouse models are in-conclusive with varying immune response consequences. SOCS1 deficient mice were shown to be hyper-responsive to LPS and have a high lethality when treated with LPS [34], [35]. In contrast to this SOCS3 deficient mice had reduced sensitivity to endotoxin shock, resulting in a high survival rate after endotoxin treatment. This was explained by an enhanced production of anti-inflammatory cytokines including IL-6, IL-11 and LIF which normally are inhibited by SOCS3 [36]. The phenotype of SOCS2 knockout mice demonstrates increased sensitivity to GH, whereas changes in immune system seem milder than with e.g. SOCS1. However, the SOCS2 knock out mouse model provides a system where both metabolic and immunological disturbances are observed. In turn this may be relevant considering the increased awareness that inflammatory disturbances are important in metabolic disorders [37].

In conclusion, we demonstrate that various TLR ligands induce SOCS2 gene expression in human DCs, and we propose that SOCS2 regulation in late phase by TLR4 signaling is dependent on an autocrine/paracrine type I IFN loop that activates SOCS2 via STAT3 and STAT5.

## Materials and Methods

### Ethics Statement

The work was approved by the Karolinska Institutet ethics committees. The informed consent from all participants was obtained and written for used human buffy coat (permit nr: 2008/2017–31). All animal experimentations were conducted in accordance with European and Swedish laws and regulation (permit nr: N415/08).

### Cell culture media, Cytokines and Reagents

L-glutamine, penicillin, streptomycin and FCS (Hyclone, Logan, UT); Ficoll-Paque (Pharmacia Biotech, Uppsala, Sweden); RPMI-1640 cell culture medium (Sigma-Aldrich, St Louis, USA); Recombinant human GM-CSF and recombinant human IL-4 (Invitrogen Biosource, Camarillo, CA); and CD14^+^ Human monocyte isolation kit II (Miltenyi Biotec, Bergisch Gladbach, Germany) were purchased from the sources as indicated for human moDCs culture. Recombinant human cytokines IFNα, IFNβ1a, IFNγ (R&D systems) and IFNλ1 (PeproTech) were used at optimal concentration of 1000 U/ml, 1000 U/ml, 20 ng/ml and 3 nM, respectively. Mouse anti-human IFNAR2 neutralizing antibodies (CD118; PBL Biomedical Laboratories, NJ, USA) and mouse IgG2a isotype controls (R&D systems, USA) were used at the concentration of 30 µg/ml.

Human moDCs were activated with the TLR2 ligand, 10 µg/ml Pam3CSK4 (a synthetic bacterial tripalmitoylated lipopeptide); TLR3 ligand, 30 µg/ml polyI:C (a synthetic analog of the dsRNA); TLR5 ligand, 1 µg/ml purified flagellin (from S. typhimurium); TLR7 ligand, 5 µg/ml imiquimod (small synthetic antiviral molecule); TLR8 ligand, 5 µg/ml ssRNA40/LyoVec (single-strand GU-rich oligonucleotide complexed with Lyo Vec); TLR9 ligand, 5 µg/ml ODN 2336 (type A CpG oligonucleotide) that were from InvivoGen (San Diego, CA, USA), and TLR4 ligand, 200 ng/ml LPS (derived from Escherchia coli O26: B6) that was from Sigma-Aldrich (St Louis, USA). L-glutamine, penicillin, streptomycin, FCS, Hepes and Dulbecco's modified Eagle's medium (DMEM) (Sigma, St Louis, MO) were used for mouse cell culture.

### Mice

Mutant mouse strains with genomic deficiency in *MyD88* [38] and *IRF3* [39] were generated by homologous recombination in embryonic stem cells as previously described. Animals were bred and kept under specific pathogen-free conditions. Mice of the C57BL/6 background were used as controls.

### Generation of human monocyte-derived DCs

DCs were generated as previously described [17]. Briefly, human PBMCs were isolated from fresh heparinized buffy coats (Department of Transfusion Medicine, Karolinska University Hospital) by Ficoll-Paque density gradient centrifugation. Human monocytes (>95% CD14+) were negatively selected by magnetic associated cell sorting (MACS) and subsequently cultured (10^6^ cells/ml) in RPMI 1640 medium supplemented with 10% FCS, 50 ng/ml GM-CSF and 20 ng/ml IL-4 for 6 days. The cells were fed with fresh medium on days 2 and 4. Immature DCs (1×10^6^/ml) cultivated for 6 days as described above were incubated with TLR ligands for different periods after an initial incubation for 1 h in fresh supplemented RPMI 1640 medium or used for siRNAs silencing experiments.

### Mouse BM-derived Macrophages

Mouse BMMs were obtained from 6 to 10 week-old mice. Mice were euthanized and the femur and tibia of the hind legs were dissected. Bone marrow cavities were flushed with 5 ml cold, sterile PBS. The bone marrow cells were washed and resuspended in DMEM medium containing glucose and supplemented with 2 mM L-glutamine, 10% FCS, 10 mM Hepes, 100 µg/ml streptomycin, 100 U/ml penicillin, and 20 to 30% L929 cell-conditioned medium (as a source of macrophage-colony stimulating factor). Bone marrow cells were passed through a 70 µm cell strainer, plated and incubated for 6 days at 37°C, 5% CO_2_. BMM cultures were then washed vigorously to remove non-adherent cells, trypsinized, counted and cultured for one day at 37°C in 6 well plates. We have previously shown by immunofluorescence staining that these BMM are F4/80+, CD14+ and Mac-3+ [40].

### Infection for mouse bone marrow-derived macrophages

*Mycobacterium bovis* BCG Montreal was grown in Middlebrook 7H9 (Difco, Carlsbad, CA) supplemented with albumin, dextrose, catalase and 50 µg/ml hygromycin. BCG was grown to mid-log phase, filtered through 40 µm cell strainers and the bacterial concentration determined by spectrophotometry. BMMs were infected at the MOI 5 and samples taken at the indicated time points.

### Transfection

Transfection of immature DCs (iDCs) was performed as previously described [17] using a commercial kit and a nucleofector machine (Amaxa Co., Köln, Germany). According to the manufacturer's instruction, the iDCs were collected on day 6. 10^7^ cells were resuspended in 100 µl human DC nucleofection solution. Small interfering RNAs (siRNA) (Qiagen, target sequences in Table 1) were added, and the mixed samples were transferred into certified cuvettes and transfected by using program U002. 500 µl pre-warmed RPMI 1640 medium, supplemented with 10% FCS, L-glutamine, penicillin and streptomycin, was added to each cuvette after transfection. The transfected cells were collected and seeded into wells of 6-well plates containing supplemented RPMI 1640 medium with 50 ng/ml GM-CSF and 20 ng/ml IL-4. After 24 hours, the cells were washed and divided into several dishes for stimulation and analysis.

**Table 1:** Target sequences for siRNA.

**NF-κB**	AAGATCAATGGCTACACAGGA
**IRF1**	CAGCCGAGATGCTAAGAGCAA
**IRF3**	CCGCTCTGCCCTCAACCGCAA
**STAT3**	CAGCCTCTCTGCAGAATTCAA
**STAT5**	CCGAGCGAGATTGTAAACCAT
**STAT6**	ACGGATAGGCAGGAACATACA
**Negative Control siRNA**	AATTCTCCGAACGTGTCACGT

### Quantitative RT-PCR

Total RNA was extracted using TRIzol (Invitrogen). Total RNA (1–3 µg) was treated with DNase I (Promega, Madison, WI) and then reverse-transcribed with a cDNA synthesis kit (Bio-Rad, Hercules, CA). The synthesized cDNA was used as template in a real-time PCR mix according to the manufacturer's standard protocol (iQ SYBR Green supermix reagents). The reactions were performed in a total volume of 20 µl with 2 µl of respective cDNA sample (7500 fast real-time PCR system, Applied Biosystems). As a control for the specificity of the real-time PCR a sample without template was included. All the measurements were performed in triplicates for each sample; the relative amounts of mRNA were calculated with the comparative threshold (Ct) method and normalized against human RP-II or mouse GAPDH. All primer sequences are provided in Table 2.

**Table 2:** Primers for real-time PCR amplification.

	Forward (5′-3′)	Reverse (5′-3′)
**Human**		
**SOCS2**	GAGCTCGGTCAGACAGGATG	AGTTGGTCCAGCTGATGTTTT
**NF-κB**	CCCCACGAGCTTGTAGGAAAG	CCAGGTTCTGGAAACTGTGGAT
**IRF1**	TGCCTCCTGGGAAGATGA	CCTGGGATTGGTGTTATGC
**IRF3**	ACCAGCCGTGGACCAAGAG	TACCAAGGCCCTGAGGCAC
**IFNα**	AGCCATCTCTGTCCTCCATGA	CATGATTTCTGCTCTGACAACC
**IFNβ**	GATTCCTACAAAGAAGCAGCAA	CAAAGTTCATCCTGTCCTTGAG
**IFNγ**	GCAGGTCATTCAGATGTAGCGG	TGTCTTCCTTGATGGTCTCCACAC
**IFNλ1**	GTGGTGCTGGTGACTTTGG	CTCCTGTGGTGACAGAGATTTG
**STAT3**	GGCCCCTCGTCATCAAGA	TTTGACCAGCAACCTGACTTTAGT
**STAT5**	GTCACGCAGGACACAGAGAA	CCTCCAGAGACACCTGCTTC
**STAT6**	CCTCGTCACCAGTTGCTT	TCCAGTGCTTTCTGCTCC
**RPII**	GCACCACGTCCAATGACAT	GTGCGGCTGCTTCCATAA
**Mouse**		
**SOCS2**	TCCAGATGTGCAAGGATAAACG	AGGTACAGGTGAACAGTCCCATT
**GAPDH**	TTGTCAAGCTCATTTCCTGGT	TTACTCCTTGGAGGCCATGTA

### Western blot analysis for SOCS2 protein or related transcriptional factors nuclear translocation

After stimulation, DCs were washed once with cold PBS. Total protein was extracted with radio immune precipitation assay (RIPA) buffer and incubated on ice for 30 minutes. Nuclear and cytoplasmic proteins were extracted using a commercial nuclear and cytoplasmic protein extraction kit (Pierce Biotechnology, Rockford, IL) according to the manufacturer's instruction. A protease inhibitor cocktail solution [Roche, Penzberg, Germany] was added prior to usage.

The cell lysates (10–30 µg per lane) were submitted to SDS-polyacrylamide gel electrophoresis (PAGE) (on 8–12% gels), transferred to polyvinylidenediflouride (PVDF) membranes for Western Blot analysis. After blocking with 5% fat-free milk dissolved in TBS-T for 1 h at room temperature, membranes were incubated over night with antibodies raised against total STAT3, STAT5, STAT6, IRF3 and SOCS2 (Cell Signaling Technology, Beverly, MA) and total p65NF-κB (BD Biosciences, Heidelberg, Germany), IRF1 (Santa Cruz biotechnology, CA, USA) respectively according to the manufacturer's instructions. Binding of these primary antibodies was visualized with goat anti-rabbit/anti-mouse immunoglobulin coupled to horseradish peroxidase (Santa Cruz biotechnology, CA, USA). After stripping, the membranes were incubated and re-probed for new antibodies. Measurement of Lamin A and β-actin proteins served as loading controls.

### Bioinformatics and Statistical analysis

Genomic sequence of human SOCS2 promoters was obtained from the UCSC Genome Bioinformatics Site (http://genome.ucsc.edu). The prediction of transcription factor binding sites in human SOCS2 promoters was performed using the binding sites searching software (http://www.genomatix.de) for the STAT family binding sites. Statistical comparisons between groups were made by analysis of variance followed by a paired *t* test or student's *t* test. Statistical significance is indicated in the figures (*P<0.05; **P<0.01).

## Supporting Information

Figure S1 **Prediction of putative SOCS2 gene promoter binding transcription factors.** (A) Diagram of the human SOCS2 gene showing the position of the different promoter regions and putative transcription factor binding sites. The thin solid line represents the genomic regions containing the SOCS2 gene. Intermediary lines represent the locations of exons, whereas the thick lines represent the translated regions. (B) Predicted transcriptional factor binding sites in the SOCS2 gene promoter regions.
(EPS)

Figure S2 **Illustration of the proposed mechanism for SOCS2 transcriptional regulation in TLR4 signaling.** LPS binds to the TLR4 on cell surface, and activates transcription factors IRF1 and IRF3 which translocate into nucleus and cause secretion of type I IFN. Autocrine-paracrine type I IFN activates a subsequent signaling by the IFNAR and leads to translocation of STATs, particularly STAT3 and STAT5 for increased SOCS2 expression.
(EPS)
